# Subretinal abscess: causative pathogens, clinical features and management

**DOI:** 10.1186/s12348-022-00315-0

**Published:** 2022-11-21

**Authors:** Beatrice Gallo, Ilaria Testi, Carlos Pavesio

**Affiliations:** 1 Uveitis Service, Moorfields Eye Hospital NHS Foundation Trust, City Road, London, EC1V 2PD UK; 2 NIHR Biomedical Research Centre for Ophthalmology at Moorfields Eye Hospital and University College London Institute of Ophthalmology, London, UK

**Keywords:** Subretinal abscess, Endogenous endophthalmitis, Therapeutic strategy, Systemic antibiotics

## Abstract

**Purpose:**

To review the literature on endogenous subretinal abscess (SRA).

**Methods:**

We searched in the literature for the terms ‘subretinal abscess’, ‘chorio-retinal abscess’ and ‘choroidal abscess’.

**Results:**

A total of 122 patients were identified, of whom 20 patients (22 eyes) had no identified systemic infective foci (group 1) and 102 (120 eyes) had systemic infective foci (group 2). The mean age for group 1 was 44.6 years (range 2 weeks-82 years) and for group 2 was 43.2 years (range 1–89 years). The responsible pathogen was identified in 90% and 95% of cases, respectively. In group 1 the most frequent causative agents were *Aspergillus* and *Nocardia*, while in group 2 were *Nocardia*, *Mycobacterium Tuberculosis* and *Klebsiella*. In both groups the most common symptoms were reduced vision (70% and 72.5%, respectively), pain (65% and 29.4%, respectively) and redness (35% and 17.6%, respectively). For group 1 there was no difference between mean initial and final visual acuity (1.7 logMAR, range 0–3 logMAR), while for group 2 mean initial and final visual acuities were 0.8 logMAR and 0.6 logMAR, respectively. Final visual acuity was significantly better in group 2 (*p* = 0.003). Anterior segment inflammation was seen in 77.3% of cases of group 1 and 66.7% of cases of group 2. In both groups the abscess most common locations were posterior pole (45.4% and 32.5%, respectively) and temporal periphery (13.6% and 13.3%, respectively). Clinical features included hemorrhages (76.5% and 76.3%, respectively) and subretinal fluid (75% in both groups). Diabetes mellitus (20% and 25.5%) and immunosuppressive drug intake (35% and 23.5%) were the main predisposing factors for SRA. Combination of systemic and intravitreal antibiotics/antifungals and vitrectomy was the main therapeutic strategy for both groups. Systemic treatment alone was used mainly for cases of tubercular etiology. The timing of vitrectomy differed between the two groups, as it more commonly followed the use of systemic and intravitreal antibiotics in the forms associated with systemic infective foci. Additional abscess drainage or intralesional antibiotics were performed in 23.8% of cases.

**Conclusion:**

At present no guideline exists for the treatment of subretinal abscess. Systemic broad-spectrum antibiotic treatment is of primary importance and should be used in all cases unless contraindicated. Combination of systemic and local treatment is the most frequently adopted strategy.

## Introduction

Subretinal abscess (SRA) is a rare and sight-threatening manifestation of endogenous endophthalmitis, where a pathogen reaches the choroid via the bloodstream from another site of the body and crosses the blood-retinal barrier, invading the retina and potentially the vitreous cavity [1]. In 1983, Wilmarth was the first to describe a case of SRA associated with endogenous endophthalmitis caused by *Aspergillus fumigatus* in an intravenous (IV) drug user [2]. Subsequently, several cases of SRA [3–5], intraretinal abscess [6], septic retinal cyst [7], choroidal or chorio-retinal abscess [8, 9] were reported, but the distinction between these entities remains unclear. Given the rarity, there are no data on the incidence of SRA. In the British Ophthalmological Surveillance Unit (BOSU) study on 62 cases of endogenous endophthalmitis, SRA was the second most common retinal finding (6.5%) after retinitis (31%) and followed by Roth’s spots (4.8%) [10]. Predisposing conditions of SRA are the same of endogenous endophthalmitis [1], including diabetes, immunosuppression, extraocular foci of infection, sepsis [4], IV drug use, blood malignancies and autoimmune diseases. The etiology is most frequently bacterial, the commonest being *Nocardia* followed by *Pseudomonas Aeruginosa*, *Streptococcus Viridans* and *Klebsiella Pneumoniae*. *Aspergillus* is the most frequent fungal agent [3]. Rarely the etiology is protozoan [11] or mixed [12]. The visual prognosis of SRA is often very poor due to the aggressive course despite treatment, with the most severe cases complicated by subretinal pseudo-hypopyon [11] and exudative or rhegmatogenous retinal detachment, requiring enucleation or evisceration if not timely treated.

We reviewed all cases of SRA with and without systemic infective foci published between 1967 and 2021, focusing on epidemiology, causative pathogen and method of identification, symptoms and signs at presentation, systemic predisposing factors and treatment approach.

## Methods

We identified published studies from Pubmed (National Library of Medicine), EMBASE (Embase.com) and Scopus (Elsevier) from inception to January 2021. The publication period ranges between September 1967 and January 2021.

There were no language restrictions. To ensure appropriate study inclusion, the search terms were ‘subretinal abscess’, ‘chorio-retinal abscess’ and ‘choroidal abscess’. The titles and abstracts were screened and full-texts were obtained for inclusion and data collection. A total of 105 articles were preliminarily enrolled and after a full-text review a total of 96 articles were chosen for inclusion, with 122 patients in total. For the rarity of the condition all chosen articles were case reports (84) and case series (12), and were considered of sufficient quality for inclusion if documented: 1) presenting clinical features, responsible pathogen and method of identification; 2) treatment strategies; 3) final outcomes. Articles lacking detailed information and cases of exogenous SRA developing after ocular surgery or ocular trauma were excluded. For the purpose of our study SRA cases without identified systemic infective foci (group 1) and with systemic infective foci (group 2) were analyzed separately. Snellen visual acuity (VA) was converted to logarithm of minimal angle of resolution (logMAR) and was analyzed as a continuous variable. Continuous variables (e.g. VA) were compared using an unpaired t-test. A loss of 0.3 logMAR of VA or more from baseline was considered a worsening, while a gain of 0.3 logMAR or more was considered an improvement.

## Results

### Group 1: SRA without identified systemic infective foci

#### Demographics

Our literature search identified 20 patients (22 eyes) with a mean age at presentation of 44.6 years (range 2 weeks-82 years). There were 11 males and 9 females. The majority of reports were from the United States (10 patients) and India (5 patients). Mean follow-up duration was 9.8 (median 6.5 , range 0.3–48 ) months.

General health was reported as unremarkable in 5 patients (25%), whereas 7 patients (35%) were on systemic steroids and/or immunosuppressive drugs, 4 (20%) were type 2 diabetic, 2 (10%) had a history of IV drug use and 2 (10%) were human immunodeficiency virus (HIV) positive. In all patients an active systemic infective process was not identified, but one suffered with gastro-enteritis 2 weeks before the onset of ocular symptoms, one had an urinary tract infection a month before and one had fevers of unknown origin during a recent exotic travel.

#### Clinical features at presentation

At presentation SRA was isolated, without vitreous involvement, in 4 patients (4 eyes) and associated with endophthalmitis in 16 patients (18 eyes). There was no difference in laterality (9 right eyes, 9 left eyes, 2 bilateral). The three most frequent symptoms were reduced vision (14 patients, 70%), pain (13 patients, 65%) and redness (7 patients, 35%). Mean symptom duration, specified for 17 patients, was 18.1 (median 7, range 1–180) days.

VA at presentation, available for 21 eyes, was 20/40 or better in 7/22 eyes (31.8%), and 14 eyes (63.6%) had a VA of 20/200 or worse. In one eye (4.6%) of a newborn baby the initial VA was not specified.

No difference was observed between initial and final VA, as the mean VA at presentation was 1.7 logMAR (median 1.9 logMAR, range: 0–3 logMAR) and the mean final VA, available for 17 eyes, was 1.7 logMAR (median 1.9 logMAR, range: 0–3 logMAR). Four eyes were enucleated.

Anterior segment involvement was observed in 17 eyes (77.3%), and signs consisted in cellularity in the anterior chamber (15 eyes), conjunctival injection (10 eyes) and hypopyon (6 eyes). In one case the anterior segment description was missing.

The most frequent location of SRA was the posterior pole (10 eyes, 45.5%), followed by the temporal periphery (3 eyes, 13.6%). Where described, hemorrhages were associated with SRA in 13 out of 17 eyes (76.5%), and subretinal fluid in 9 out of 12 eyes (75%).

#### Causative pathogens and methods of identification

Causative agents were identified in 18/20 patients (90%), and the most frequent were *Aspergillus* (*Fumigatus*, *Nidulans*, *Terreus* and *Flavus* species) and *Nocardia*, detected in 4 and 3 patients, respectively. Given the absence of a systemic infective focus, pathogen identification was from ocular samples, the commonest being SRA (8 eyes from 8 patients), followed by vitreous (7 eyes from 7 patients). In the 3 patients (3 eyes) with isolated SRA where the vitreous analysis was performed it did not allow the pathogen identification, while of the 16 patients (18 eyes) with SRA associated with vitritis the pathogen was isolated from SRA drainage or biopsy in 7 eyes (7 patients) and from vitreous in 7 eyes (7 patients). In the 8 eyes (8 patients) where the vitreous culture failed in yielding a growth the pathogen was identified by direct drainage of the SRA.

Clinical features, pathogen and method of identification of SRA without systemic foci are summarized in Table 1.

**Table 1:** Summary of study details, clinical features, pathogen and method of identification of SRA without systemic foci

S/N	Study	Year	Country	N of patients	Laterality	Sex	Age	Type of SRA	Co-morbidities	Causative pathogen	Method of identification
1	Wilmarth, Annal Ophthal	1983	USA	1	RE	M	27	SRA + EE	IV drug and amphetamins use	*Aspergillus Fumigatus*	vitreous, cotton wool balls
2	Halperin, Arch Ophthal	1988	USA	1	RE	M	40	SRA + EE	IV drug use	*Aspergillus Flavus*	SRA
3	Shields, Retina	1995	USA	2	LE	F	6	SRA + EE	none	not isolated	–
					LE	F	2 weeks	SRA + EE	none	not isolated	–
4	Garg, Retina	2006	USA	1	RE	F	52	SRA + EE	DM	*Moraxella* spp.	vitreous
5	Huynh, Ret Cas Brief Rep	2008	USA	1	BE	F	62	SRA + EE SRA + EE	auto-immune haemolitic anaemia	*Candida A.*	SRA
6	Kanuraki, Int Ophthalmol	2010	Japan	1	RE	F	51	SRA	cirrhosis, liver transplant	*Candida A.*	epiretinal proliferative tissue
7	Anderson, Ret Cas Brief Rep	2012	USA	1	LE	M	40	SRA	none	*Acanthamoeba*	SRA
8	Matthews, Indian J Ophthalmol	2013	UK	1	RE	M	67	SRA + EE	rheumatoid arthritis	not specified fungus (phaeohyphomycosis)	SRA
9	Panigrahi, Indian J Ophthalmol	2014	India	1	RE	M	50	SRA + EE	healthy	*Aspergillus Terreus*	vitreous
10	Silva, Retina	2015	USA	2	RE	M	46	SRA + EE	HIV	*Nocardia Asteroides*	SRA
					LE	F	69	SRA	Wegener granulomatosis	*Nocardia Asteroides*	enucleated eye
11	Cheng, Ret Cas Brief Rep	2016	Australia	1	LE	M	82	SRA + EE	DM	*E. coli*	SRA
12	Xu, BMC Ophthalmol	2018	China	1	RE	M	61	SRA + EE	DM, peptic ulcer	*Klebsiella P.*	vitreous
13	Joseph, Indian J Ophthalmol	2018	India	1	RE	M	36	SRA + EE	HIV	*Cryptococcus Neoformans*	vitreous
14	Majumder, Ocul Immun Inflamm	2018	India	1	BE	M	25	SRA (RE) SRA + EE (LE)	glomerolusclerosis	*Nocardia Arthritidis*, *Mycobacterium TB*	SRA
15	Verma, Ocul Immun Inflamm	2020	India	1	LE	M	45	SRA + EE	none	*Citrobacter*	vitreous
16	Nair, Indian J Ophthalmol	2020	India	1	LE	F	14	SRA + EE	none	*Mycobacterium TB*	acqueous
17	Mata-Moret, Europ J Ophthalmol	2020	Spain	1	LE	F	42	SRA + EE	asthma	*Aspergillus Nidulans*	vitreous
18	Yang, Ret Cas Brief Rep	2021	USA	1	LE	F	77	SRA + EE	DM	*S. aureus*	SRA

*S/N* Study number, *RE* Right eye, *LE* Left eye, *BE* Both eyes, *M* Male, *F* Female, *SRA* Subretinal abscess, *EE* Endogenous endophthalmitis, *IV* Intravenous, *DM* Diabetes mellitus

#### Therapeutic approaches

The most common therapeutic strategy was the combination of systemic and intravitreal antibiotics or antifungals followed by vitrectomy (13 patients, 13 eyes; 65%). In the majority of these patients (9 patients, 9 eyes, 69.2%) vitrectomy was a second line treatment performed for progression despite systemic and intravitreal antibiotics/antifungals, and in 4 patients (4 eyes, 30.8%) was first line strategy with the dual purpose of diagnosis and treatment. Additional SRA drainage was performed in 5 patients (5 eyes) and intralesional antibiotics or antifungals were administered in 3 patients (3 eyes). In one case (one eye) enucleation was performed due to failure of these treatments.

Vitrectomy and SRA drainage without systemic treatment was adopted only in one eye of a case caused by *Aspergillus* where intravitreal agents were not deemed necessary for minimal vitreous involvement [13].

Systemic treatment and vitrectomy without intravitreal antibiotics was performed in 2 cases (2 eyes).

Of the 17 patients undergoing vitrectomy, in 8 it was first line treatment while in the remainder 9 it was performed as second line treatment.

Systemic treatment was the only treatment strategy in one eye of a case of Tubercular etiology [14].

Enucleation was the first line treatment for 2 paediatric cases (2 eyes) where retinoblastoma could not be clinically excluded [15] and for one case (one eye) refusing other treatments where the SRA progressed to pan-ophthalmitis [16]. Systemic and intravitreal agents were the strategy adopted for the eye of a patient with bilateral involvement by *Candida *where the fellow eye underwent vitrectomy [17] and in one case of bilateral SRA caused by *Nocardia* and *Mycobacterium TB* where the fellow eye underwent vitrectomy [18].

Steroids were used in four cases of bacterial etiology: one receiving intravitreal dexamethasone at the end of vitrectomy [19] and three receiving oral steroids, of whom one was also on systemic anti-tubercular treatment. Intravitreal dexamethasone was given in a diabetic patient with SRA caused by *S. aureus* where the final VA improved.

#### Responses to treatment, secondary complications and their treatment

Of the 17 eyes where the final VA was available, in 3 eyes (17.6%) VA was 20/40 or better, in 2 eyes (11.8%) was between 20/40 and 20/200, and in 12 eyes (70.6%) was 20/200 or worse.

Comparing initial and final VA, in 6 eyes (35.3%) VA improved, in 8 eyes (47.1%) remained stable and in 3 eyes (17.6%) worsened. VA was not specified for one eye and 4 eyes were enucleated.

In 8/13 cases multiple surgeries were necessary either because the lesion expanded despite previous vitrectomy or for development of complications such as retinal detachment or retinal traction. One case caused by *Aspergillus Fumigatus*, progressed despite the initial treatment with systemic and intravitreal amphotericin, vitrectomy and lensectomy, rendering enucleation necessary [2]. In 2 paediatric cases enucleation was performed as retinoblastoma could not be ruled out.

Baseline and final VA, treatment interventions and final outcomes of SRA without systemic foci are summarized in Table 2.

**Table 2:** Summary of initial and final visual acuity, treatment interventions and final outcomes of SRA without systemic foci

S/N	Study	Pathogen	Initial VA (logMAR)	Final VA (logMAR)	Treatment	Outcome
1	Wilmarth, Annal Ophthal	*Aspergillus Fumigatus*	1.9	–	- intravenous amphotericin - intravitreal amphotericin - vitrectomy + lensectomy - enucleation	enucleated
2	Halperin, Arch Ophthal	*Aspergillu Flavus*	1.9	1.9	vitrectomy + SRA drainage	same VA
3	Shields, Retina	not isolated	2.7	–	enucleation	enucleated
		not isolated	N/A	–	enucleation	enucleated
4	Garg, Retina	*Moraxella* spp	2.3	0.6	- vitrectomy + lensectomy - intravitreal vancomycin and ceftazidime - intravenous vancomycin, ceftazidime and ceftriaxone	better VA
5	Huynh, Ret Cas Brief Rep	*Candida A*.	0.3; 0.3	0; 0	- LE vitrectomy + SRA drainage oral voriconazole - RE 2 intravitreal amphotericin - LE 2^nd^ vitrectomy	BE better VA
6	Kanuraki, Int Ophthalmol	*Candida A*.	0	2.3	- Systemic acetylspiramycin and levofloxacin - vitrectomy + cataract + SRA drainage + silicone oil + intralesional fluconazole and imipenem - vitrectomy + silicone oil + antifungals - vitrectomy + scleral encircling + membrane removal + silicone oil + antifungals - intravitreal amphotericin - intravenous fluconazole	worse VA
7	Anderson, Ret Cas Brief Rep	*Acanthamoeba*	1.3	1.3	- systemic ceftriaxone, metronidazole, and fluconazole - vitrectomy + intravitreal vancomyin, ceftazidime and amphotericin - 2^nd^ vitrectomy + SRA drainage - systemic amphotericin, fluconazole, sulfamethoxazole, trimethoprim, rifampin	same VA
8	Matthews, Indian J Ophthalmol	phaeohyphomycosis	0.2	1.5	- topical prednisolone and cyclopentolate - pyrimethamine + sulfadiazine + clindamycin - vitrectomy - SRA biopsy + silicone oil - oral voriconazole - 2^nd^, 3^rd^ and 4^th^ vitrectomy - intravitreal amphotericin	worse VA
9	Panigrahi, Indian J Ophthalmol	*Aspergillus Terreus*	2.7	2.7	- vitrectomy + intravitreal vancomicin, ceftazidime, voriconazole - 6 x intravitreal voriconazole - 2^nd^ vitrectomy + endolaser + silicone oil - oral voriconazole	same VA
10	Silva, Retina	*Nocardia Asteroides*	1.9	1.9	- intravitreal ganciclovir - oral acyclovir, azithromycin and valganciclovir - 1^st^ vitrectomy - 2^nd^ vitrectomy + retinotomy + SRA biopsy + silicone oil - oral TMP-SMX - 2 x intravitreal amikacin - enucleation	same VA
		*Nocardia Asteroides*	0.3	–		enucleated
11	Cheng, Ret Cas Brief Rep	*E. coli*	2.3	2.3	- intravitreal vancomycin, ceftazidime, foscarnet and voriconazole - intravenous ceftriaxone and flucloxacillin - vitrectomy + AC washout + silicone oil - 2^nd^ vitrectomy + chorio-retinal biopsy - oral ciprofloxacin and amoxicillin	same VA
12	Xu, BMC Ophthalmol	*Klebsiella P*	2.7	2.3	- intravitreal vancomycin and ceftazidime - topical levofloxacin, prednisolone 1% and atropine - intravenous cefoperazone - vitrectomy + phaco	better VA
13	Joseph, Indian J Ophthalmol	*Cryptococcus Neoformans*	3.0	3.0	- vitrectomy + intravitreal ganciclovir - intravitreal amphotericin - oral valganciclovir - systemic amphotericin	same VA
14	Majumder, Ocul Immun Inflamm	*Nocardia Arthritidis*, *Mycobacterium Tubercolosis*	0.1; 1.9	0.3; 1.9	- oral ATT and steroid - LE vitrectomy + silicone oil - RE intravitreal imipenem - intravenous cefotaxime and amikacin	RE worse and LE better VA
15	Verma, Ocul Immun Inflamm	*Citrobacter*	2.3	1.0	- intravenous vancomicin and ceftriaxone - topical antibiotics, steroids and cyclopentolate - intravitreal vancomicin and ceftazidime - 1^st^ vitrectomy + intralesional piperacillin and tazobactam + silicone oil - systemic prednisolone - oral antibiotic ciprofloxacin - 2^nd^ vitrectomy + phaco + buckle + silicone oil	better VA
16	Nair, Indian J Ophthalmol	*Mycobacterium Tubercolosis*	0	N/A	- oral ATT and steroids	–
17	Mata-Moret, Europ J Ophthalmol	*Aspergillus Nidulans*	2.3	3.0	- oral voriconazole - 7 x intravitreal voriconazole - 1^st^ vitrectomy + intravitreal foscarnet - 2^nd^ vitrectomy + SRA drainage + subretinal voriconazole - 3^rd^ vitrectomy + SRA aspiration + lensectomy + endolaser	worse VA
18	Yang, Ret Cas Brief Rep	*S. aureus*	2.7	1.9	- intravenous vancomycin and ceftazidime - intravitreal vancomycin, ceftazidime and dexamethasone - 1^st^ vitrectomy - 2^nd^ vitrectomy+SRA drainage - 3^rd^ vitrectomy + silicone oil - topical prednisolone and moxifloxacin	better VA

*S/N* Study number, *RE* Right eye, *LE* Left eye, *BE* Both eyes, *M* Male, *F* Female, *N/A* Not available, *ATT* Anti-tubercular treatment

### Group 2: SRA with identified systemic infective foci

#### Demographics

One hundred and two patients (120 eyes) were identified. There were 69 males and 33 females, with a mean age of 43.2 (median 44; range 1–89) years specified for 101 patients. The mean duration of follow-up, available for 96 patients, was 8.3 (median 6, range 0.2–48) months.

Twenty-three (22.5%) patients were healthy, 26 patients (25.5%) had diabetes mellitus, 24 (23.5%) were on immunosuppressive medications or oral steroids, and 3 (2.9%) were HIV-positive. The most common infective focus was the respiratory system (25 patients) followed by disseminated infection (16 patients) and soft tissue infection (13 patients).

#### Clinical features at presentation

SRA was isolated in 13 patients (14 eyes), associated with endophthalmitis in 84 patients (100 eyes), and not better described in 5 patients (6 eyes). There were 30 right eyes, 40 left eyes, 18 bilateral and 14 eyes unilateral with unspecified laterality.

The three most frequent symptoms were reduced vision (74 patients, 72.5%), sudden in 8 patients and gradual in 66 patients, pain (30 patients, 29.4%) and redness (18 patients, 17.6%), followed by floaters (9 patients, 8.8%) and photophobia (5 patients, 4.9%).

Mean symptom duration, specified for 67 patients, was 16.3 days (median 3 days, range 0–365 days).

The mean visual acuity at presentation, available for 105 eyes, was 1.0 (median 1, range: 0–3) logMAR, and the mean final visual acuity, available for 99 eyes, was 0.6 (median 0, range: 0–3 ) logMAR.

VA at presentation, available for 105 eyes, was 20/40 or better in 15 eyes (14.3%), between 20/40 and 20/200 in 25 eyes (23.8%), and 20/200 or worse in 65 eyes (61.9%).

Anterior segment involvement was observed in 76 eyes from 68 patients (66.7%), with the most frequent signs being anterior chamber cells (63 eyes), conjunctival hyperemia/chemosis (33 eyes) and flare (23 eyes).

The most frequently observed location of SRA was the posterior pole (39 eyes, 32.5%) followed by the temporal periphery (16 eyes, 13.3%). Hemorrhages were present in 58/76 eyes (76.3%), and subretinal fluid in 33/44 eyes (75%).

#### Causative pathogens and methods of identification

The causative pathogen was identified in 97 patients (95%) and not identified in 5 patients.

*Nocardia* was the most frequent pathogen - detected in 24 patients (23.5%) - followed by *Mycobacterium Tuberculosis* (18 patients, 17.6%), *Klebsiella* (18 patients, 17.6%) and *S. aureus* (14 patients, 13.7%). *Mycobacterium Tuberculosis* and *Nocardia* spp. were the two main responsible agents of respiratory infections (14 and 5 patients, respectively) and systemic infections (3 and 14 patients, respectively).

Pathogens were identified from ocular tissues in 42 patients (43.3%) and from extra-ocular tissues in 49 patients (50.5%), of whom 27 from blood cultures. In six patients *Mycobacterium Tuberculosis* was not isolated but tubercular disease was diagnosed based on the typical pulmonary findings on chest X-ray or CT and a positive Mantoux test and/or QuantiFERON-TB gold.

The vitreous was the main ocular source for pathogen identification (20 patients, 47.6%, 20 eyes), followed by SRA (14 patients, 33.3%, 14 eyes) and aqueous (7 patients, 16.7%, 7 eyes).

In all cases of isolated SRA (13 patients, 14 eyes) the pathogen was identified, but ocular sampling was performed only in 7/13 (53.8%), with a yielding rate for vitreous of 20% (1/5 patients) and for SRA of 100% (3/3 patients).

In cases of SRA and endophthalmitis (84 patients, 100 eyes), vitreous sampling was performed in 50 cases and the pathogen yielding rate for vitreous was 38% (19 cases), while SRA biopsy allowed pathogen identification in 20% of cases (10 cases).

*Nocardia* was by far the most common pathogen in immunosuppressed patients (18/24 patients), *Klebsiella* and *S. aureus* were the main causative agents in diabetic patients, detected in 9/26 cases and 7/26 cases, respectively; *Mycobacterium Tuberculosis* was the most common pathogen in healthy subjects (12/23 patients).

Clinical features, pathogen and method of identification of SRA with systemic foci are summarized in Table 3.

**Table 3:** Summary of study details, clinical features, pathogen and method of identification of SRA with systemic foci

S/N	Study	Year	Country	N of patients	Laterality	Sex	Age	Type of SRA	Co-morbidities	Systemic infective process	Causative pathogen	Method of identification
1	Manor, Ophthalmologica	1965	Israel	1	LE	F	49	Pre-papillary abscess + EE	mitralic valve stenosis	bacterial endophthalmitis	not isolated	–
2	Davidson, Trans Am Ac Ophthalmol	1967	USA	1	LE	M	46	SRA + EE	liver gallstones	lung infection	*Nocardia Asteroides*	enucleated eye
3	Fleming, Can J Ophthalmol	1972	USA	1	BE	M	10 months	SRA (vitreous not described)	congenital small bowel atresia, deficit of growth	UTI, lung abscess	*Candida A.*	blood lung
4	Naidoff, Am J Ophthalmol	1975	USA	1	LE	M	26	SRA + EE	renal transplant	pneumonia, brain abscess	*Aspergillus Fumigatus*	lung vitreous
5	Hiss, Ophthalmology	1988	USA	1	RE	M	63	SRA + EE	DM, HBP, angina, chronic renal failure, anemia, polyarteritis nodosa	meningitis, pneumonia, sepsis	*Criptococcus Neoformans*	SRA cerebro-spinal fluid
6	Mamalis, Ann Ophthalmol	1988	USA	1	LE	M	44	SRA + EE	cardiac transplant	nocardiosis with testicular abscess	*Nocardia Asteroides*	testis
7	Gregor, Retina	1989	USA	1	RE	M	46	SRA	cardiac transplant	nocardiosis with brain abscess	*Nocardia Asteroides*	SRA
8	Coll, Retina	1994	USA	1	RE	M	44	choroidal abscess	DM, heroin use	endocarditis, toe cellulitis	*S. aureus*	blood toe
9	Webber, British J Ophthalmol	1995	UK	1	RE	M	23	SRA + EE	lung transplant	systemic	*Pseudomonas A.*	sputum SRA
10	Biswas, Retina	1995	India	2	RE	F	42	SRA + EE	sarcoidosis	lung TB	*Mycobacterium TB*	vitreous
					RE	F	58	SRA + EE	DM	systemic TB	*Mycobacterium TB*	acqueous
11	Jolly, Arch Ophthalmol	1996	Canada	1	RE	F	40	SRA + EE	renal transplant	systemic	*Nocardia Asteroides*	lung
12	Yarng, Ophthal Surg Las Im	1997	Taiwan	1	LE	M	39	SRA + EE	none	liver abscess	*Klebsiella P.*	blood liver vitreous
13	Rimpel, British J Ophthalmol	1999	USA	1	LE	M	56	SRA	multiple myeloma	endocharditis, brain septic emboli	*Streptococcus Viridans*	blood vitreous
14	Lakosha, Retina	2000	Canada	1	RE	M	41	SRA	chronic myeloid leukemia	subcutaneous abscess	*Nocardia Farcinica*	subcutaneous abscess
15	Harris, Am J Ophthalmol	2000	USA	1	RE	M	32	SRA + EE	beta-thalassemia major	liver and kidney abscess	*Klebsiella P.*	liver blood
16	Costen, Eye	2001	UK	1	BE	F	68	SRA + EE	none	meningitis, sepsis	*Streptococcus Pyogenes*	blood
17	Yao, Eur J Pediatr	2001	Taiwan	1	LE	F	14	SRA + EE	beta-thalassemia major	pneumonia, mastoiditis	*Klebsiella P.*	external auricular canal
18	Yoon, Retina	2003	Korea	2	Unilateral, side NA	M	41	SRA + EE	DM	liver abscess	*Klebsiella P.*	vitreous, blood, liver
					BE	M	47	SRA + EE	DM	liver abscess	*Klebsiella P.*	blood, liver
19	Bozbeyoglu, Retina	2004	Turkey	1	LE	M	46	choroidal abscess (vitreous not described)	renal transplant	nocardiosis with brain and lung abscess	*Nocardia Asteroides*	SRA blood
20	Shah, Indian J Ophthalmol	2004	India	1	BE	F	23	SRA + EE	none	genito-urinary tract	*Candida A.*	vitreous vagina
21	Wjjesekera, Eye	2004	UK	1	LE	M	75	SRA + EE	post TB bronchiectasis	chronic brochial colonization	*Pseudomonas A.*	SRA
22	Motley, Retina	2005	USA	1	LE	M	25	choroidal abscess + EE	cystic fibrosis	lung infection on cystic fibrosis reacutization	*Pseudomonas A.*	SRA vitreous sputum
23	Yu, Am J Neurorad	2005	Canda	1	LE	F	41	SRA + EE	bone marrow transplant	systemic	*Nocardia Asteroides*	skin
24	Rafiei, Europ J Ophthalmol	2005	USA	1	LE	M	61	SRA + EE	idiopathic thrombocytopenia purpura	systemic	*Nocardia Asteroides*	bronchus skin
25	Dodds, Ocul Imm Inflamm	2006	Argentina	1	LE	F	26	SRA + EE	SLE	lung, brain, cerebellum abscesses	*Nocardia Farcinica*	SRA
26	Yang, Ophthalmol	2007	Taiwan	1	Unilateral, side NA	M	48	SRA + EE	DM	liver abscess	*Klebsiella P.*	blood liver
27	Contreras, Ret Cas Brief Rep	2007	Spain	1	BE	M	24	SRA + EE	acute myeloid leukemia, graft versus host disease	sepsis	*Candida A.*	blood central catheter
28	Christoforidis, Ret Cas Brief Rep	2007	USA	1	RE	F	56	SRA + EE	DM, nephrolithiasis, peptic ulcer	kidney abscess	*Klebsiella P.*	blood vitreous
29	Li, Int Ophthalmol	2008	China	1	RE	M	75	choroidal abscess + EE	bronchiectasis	pneumonia	*Pseudomonas A.*	enucleated eye sputum
30	Jones, Eye	2010	UK	1	LE	F	32	SRA	bone marrow transplant (aplastic anemia)	brain, lung liver abscesses	*Nocardia Asteroides*	lymph node lung
31	Trigui, Int Ophthalmol	2011	Tunisia	1	LE	M	27	SRA + EE	DM	sepsis	*S. aureus*	skin
32	Eschle-Meniconi, Surv Ophthalmol	2011	Switzerland	1	LE	M	78	SRA + EE	prostate ca, Hodgkin lymphoma	brain multiple abscesses, UTI	*Nocardia Asteroides*	SRA
33	Gupta, Ret Cas Brief Rep	2012	USA	1	LE	M	89	chorio-retinal abscess + EE	colon ca, prostate ca, HBP	soft tissue	*Pseudomonas A.*	conjunctiva blood
34	Peeler, J Neuro-ophthalmol	2013	USA	2	BE	M	16	SRA + EE	none	sepsis with CNS infacrtions	*S. aureus*	blood, skin, perichardial fluid, hip
					RE	F	62	SRA + EE	necrotizing pancreatitis	entero-cutaneous fistula, brain abscess	*Bacillus*	blood
35	Eisenberg, Ret Cas Brief Rep	2014	USA	1	RE	M	40	SRA	acute myeloid leukemia	systemic	*Nocardia Asteroides*	skin
36	Arai, Clin Ophthalmol	2014	Japan	1	BE	M	64	SRA (vitreous not described)	rheumatoid arthritis, rectal ca, pericarditis	pneumonia	*Candida A.*	exenteratio
37	Siu, BMJ Cas Rep	2015	China	1	LE	M	43	SRA	DM	liver abscess	*Klebsiella P.*	blood
38	Shetty, Indian J Ophthalmol	2015	India	1	LE	F	33	SRA	none	lung TB	*Mycobacterium TB*	a
39	Silva, Retina	2015	USA	3	RE	M	45	SRA	acute lymphoblastic leukemia	pneumonia	*Nocardia Cyriacigeorgica*	SRA
					LE	F	51	SRA + EE	SLE	systemic	*Nocardia Farcinica*	SRA
					LE	F	21	SRA	IgA nephropathy	lung abscess	*Nocardia Farcinica*	SRA
40	Won Jin, Optom Vis Sci	2015	Korea	1	BE	M	59	SRA + EE	none	prostate abscess	*Klebsiella P.*	vitreous
41	Richards, Clin Exp Ophthalmol	2015	Australia	1	BE	M	80	SRA + EE	DM, previous syphilis, hepatitis B	liver and cerebral abscesses	*Nocardia Beijingensis*	SRA
42	Tsai, BMC Ophthalmol	2015	Taiwan	1	LE	M	56	SRA + EE	DM	liver and subdural abscess	not isolated	
43	Kamath, BMK	2016	India	1	RE	M	28	SRA + EE	TB, DM	muscle abscess	*Mycobacterium TB*	muscle
44	Schlaenm Ret Cas Brief Rep	2016	Argentina	1	RE	M	47	SRA + EE	acute myeloid leukemia	sepsis	*Fusarium Solani*	vitreous
45	Venkatesh, Int J Ret Vitr	2016	India	1	LE	F	30	SRA + EE	none	cellulitis	not isolated	–
46	Soria, Cas Rep Ophthalmol	2016	Argentina	1	LE	M	24	SRA (vitreous not described)	none	miliary TB	*Mycobacterium TB*	lymph node
47	Martel, J Fran Ophtalmol	2017	France	1	LE	M	60	SRA + EE	DM	liver and UTI	*Klebsiella P.*	blood urine
48	Ganesh, Indian J Ophthalmol	2017	India	1	BE	M	37	SRA + EE	none	lung TB	*Mycobacterium TB*	vitreous
49	Kimura, Cas Rep Ophthal	2017	Japan	1	BE	M	62	SRA + EE	hepatitis, liver abscess, spondylosis, disseminated intravascular coagulation		*Klebsiella P.*	liver abscess blood
50	Boonsopon, J Med Cas Rep	2017	Thailand	1	RE	F	29	SRA + EE	HIV	lung TB	*Mycobacterium TB*	conjunctiva
51	Pittenger, BMJ	2017	USA	1	RE	M	32	SRA (vitreous not described)	IV drug use	endocharditis	*S. aureus*	blood
52	Fortun, Ophthal Surg Las	2017	USA	7	RE	F	14	SRA	healthy	myositis, ostemyelitis	*S. aureus* (all)	blood
					BE	M	32	SRA + EE	HIV	cellulitis		skin
					LE	M	47	SRA + EE	DM	cellulitis, osteomyelitis		toe
					RE	M	78	SRA + EE	DM	cellulitis		sacral abscess
					BE	F	62	SRA + EE	breast ca	sepsis		blood
					BE	M	64	SRA + EE	DM	ostemyelitis		finger
					BE	F	51	SRA + EE	DM	paraspinal abscess,		blood
53	Oduard, Clin Exp Ophthalmol	2017	Australia	2	RE	M	58	SRA + EE	DM, HBP, hypercholesterolemia	hepatic abscess	*Klebsiella P.*	blood, liver
					BE	M	51	SRA + EE	hypercholesterolemia	hepatic abscess	*Klebsiella P.*	liver, urine
54	Pappuru, Int Ophthalmol	2017	India	1	LE	F	26	SRA	none	lung TB	*Mycobacterium TB*	a
55	Bendhe, J Ophthal Inflamm Infect	2017	India	1	LE	M	74	SRA + EE	DM	UTI, septicemia	*Roseomonas mucosa*	SRA
56	Dutta-Majumder, Ocul Imm Inflamm	2018	India	12	Unilateral, side NA	F	25	SRA + EE	healthy	TB	*Mycobacterium TB* (all)	acqueous
					Unilateral, side NA	M	14	SRA + EE	lung TB	lung TB		acqueous
					Unilateral, side NA	M	45	SRA + EE	healthy	TB		acqueous, vitreous
					Unilateral, side NA	M	22	SRA + EE	lung TB	lung TB		a
					Unilateral, side NA	M	23	SRA + EE	lung TB	lung TB		a
					Unilateral, side NA	M	15	SRA + EE	healthy	lung TB		acqueous
					Unilateral, side NA	F	26	SRA + EE	lung TB	lung TB		vitreous
					Unilateral, side NA	F	17	SRA + EE	lung TB	lung TB		vitreous
					Unilateral, side NA	F	19	SRA + EE	healthy	TB		acqueous
					Unilateral, side NA	M	29	SRA + EE	healthy	TB		acqueous, vitreous
					Unilateral, side NA	M	62	SRA + EE	healthy	lung TB		a
					Unilateral, side NA	F	60	SRA + EE	healthy	lung TB		a
57	Harvey, BMJ Cas rep	2018	UK	1	LE	M	26	SRA + EE	DM	sepsis, muscle abscess	*S. aureus*	blood
58	Prajapati, BMJ Cas Rep	2018	UK	1	RE	M	–	SRA + EE	HIV	glomerulonephritis, sepsis	*S. aureus*	blood
59	Zafar, BMC Res Not	2018	Pakistan	1	RE	F	32	SRA + EE	none	vaginal infection	*Candida A.*	vitreous
60	Chawla, Middle East Afr J Ophthalmol	2018	India	1	BE	M	47	SRA + EE	none	lung TB	*Mycobacterium TB*	cervical lymph node
61	Puri, Am J Ophthalmol	2018	USA	1	RE	F	49	SRA + EE	bullous pemphigoid	systemic	*Nocardia Farcinica*	brain
62	Xu, BMC Ophthalmol	2018	China	1	LE	M	58	SRA + EE	DM, nephrotic syndrome	pneumonia with lung abscess	*Nocardia*	blood sputum
63	Tran, Clin Exp Ophthal	2019	Australia	1	LE	M	37	SRA + EE	Hodgkin’s Lymphoma	systemic	*Nocardia Farcinica*	brain
64	Scavelli, Am J Cas Rep	2019	USA	1	LE	F	25	SRA + EE	SLE	systemic	*Nocardia Farcinica*	blood
65	Manoharam, JRSM Open	2019	UK	1	LE	M	41	SRA + EE	chronic pancreatitis, vitamin D deficiency	splenic abscess, sepsis	*Proteus Mirabilis*, *Enterococcus Faecium*, *E. coli*	blood
66	Mohd-Ilham, Cureus	2019	Malaysia	1	RE	F	39	SRA + EE	DM, recurrent UTI	Pyelonephritis, sepsis	*Klebsiella P.*	blood
67	Angermann, Ocul Imm Inflamm	2019	Austria	1	LE	M	56	SRA + EE	brain astrocytoma	systemic	*Nocardia Cyriacigeorgica*	vitreous SRA
68	Dogra, Ocul Imm Inflamm	2020	India	1	LE	M	48	SRA + EE	hepatitis C, liver cirrhosis	UTI	*Klebsiella P.*	urine vitreous
69	Yiesiltas, Ocular Imm Inflamm	2020	Turkey	1	LE	M	40	SRA + EE	none	onychomycosis	*Candida A.*	vitreous
70	Shen, Retina	2020	Canada	1	RE	M	28	SRA (vitreous not described)	IV drug use	Endocarditis, lung septic emboli, MRSA bacteremia	*Klebsiella P.*	blood
71	Hojjatie, J Ophthalmic Inflamm Infect	2020	USA	1	RE	M	64	SRA + EE	liver transplant	pneumonia	*Nocardia* *Farcinica*	BAL
72	Vamsidhar, J R Coll Physicians Edinb	2020	India	1	BE	M	31	SRA (vitreous not described)	none	systemic	*S. aureus*	blood muscle
73	Lim, Case Rep Ophthalmol Med	2020	Korea	2	LE	F	50	SRA + EE	DM	liver abscess	*Klebsiella P*.	blood, liver
					RE	F	62	SRA + EE	DM	liver abscess	not isolated	–
74	Nair, Indian J Ophthalmol	2020	India	1	RE	M	25	SRA + EE	none	systemic	*Mycobacterium TB*	lung
75	Kapoor, Indian J Ophthalmol	2020	India	1	LE	F	80	SRA + EE	DM, HBP	perinephric abscess	not isolated	–
76	Malik, BMJ Cas Rep	2020	Pakistan	1	RE	M	50	SRA + EE	demyelinating polyneuropathy	systemic	*Nocardia*	blood BAL
77	Fan, Ret Cas Brief Rep	2020	USA	1	LE	M	46	SRA + EE	none	liver and splenic abscess	*Klebsiella P.*	vitreous
78	Cunha, Ret Cas Brief Rep	2021	Portugal	1	LE	M	50	SRA	lung silicosis	systemic	*Nocardia Abscessus*	bronchus

*S/N* Study number, *RE* Right eye, *LE* Left eye, *BE* Both eyes, *M* Male, *F* Female, *SRA* Subretinal abscess, *EE* Endogenous endophthalmitis, *IV* Intravenous, *DM* Diabetes mellitus, *UTI* Urinary tract infection, *HBP* High blood pressure, *TB* Tuberculosis, *SLE* Systemic lupus erythematosus, *ca* Cancer, *CNS* Central nervous system

^a^Diagnosis based on typical imaging findings and positive Q-gold test

#### Therapeutic approaches

The combination of systemic and intravitreal antibiotics/antifungals with vitrectomy was the most common therapeutic approach, performed in 28 patients (27.5%), with additional drainage of SRA and/or intralesional antibiotics or antifungals performed in most of cases (21 patients and 1 patient, respectively).

Vitrectomy was the first line strategy in 6 patients, all caused by *Klebsiella* or *Nocardia*, while for the remainder (33 patients) it was performed as second line treatment for failure of other treatment modalities. In 10 cases multiple surgeries were necessary to address SRA progression or complications (retinal detachment or tractional complications).

Combined systemic and intravitreal antibiotics were the second most common treatment strategy (20 patients). In a limited number of cases (3 cases) only vitrectomy and intravitreal antibiotics were adopted. Intravitreal dexamethasone was used in 9 eyes, of whom 7 eyes had SRA caused by *Klebsiella*, one eye caused by *Mycobacterium TB* and in one eye the pathogen was not isolated. Of these, in 6 eyes the VA improved, in one eye the VA remained the same, in one eye VA not specified and in one eye evisceration was necessary.

Systemic treatment without other treatment modalities was used in 18 patients, of whom 6 had TB, 5 had *S. aureus*-related and 4 had *Nocardia*-related infections.

Combined systemic and topical antibiotics or steroids were adopted in 18 cases, the majority of whom (13/18) had TB.

In one SRA case caused by *Klebsiella* treatment consisted only in intravitreal antibiotics, and one case caused by *Nocardia* received systemic treatment and intravitreal anti-VEGF.

In 13 cases enucleation (7 cases), evisceration (4) or exenteration (2 cases) were performed due to failure of other treatments.

#### Responses to treatment, secondary complications and their treatment

Of the 99 eyes where the final VA was available, in 29 eyes (29.3%) was 20/40 or better, in 30 eyes (30.3%) was between 20/40 and 20/200, and in 40 eyes (40.4%) was 20/200 or worse. Comparing initial and final VA of 89 eyes, in 49 eyes (55.1%) VA improved, in 23 eyes (25.8%) remained stable and in 17 eyes (19.1%) worsened. Final VA was not specified in 9 eyes. Six eyes were enucleated, 5 were eviscerated and 1 was exenterated.

In 10 cases multiple surgeries were necessary either because the lesion expanded despite the previous surgery or for development of complications such as retinal detachment or retinal traction. Twelve cases were non responsive to treatment and therefore enucleation, evisceration or exenteration were necessary, and the most common pathogens associated with these were *Nocardia* (one evisceration and two enucleations) and *Pseudomonas* (one evisceration and two enucleations).

Baseline and final VA, treatment interventions and final outcomes of SRA with systemic foci are summarized in Table 4.

**Table 4:** Summary of initial and final visual acuity, treatment interventions and final outcomes of SRA with systemic foci

S/N	Study	Pathogen	Initial VA (logMAR)	Final VA (logMAR)	Treatment	Outcome
1	Manor, Ophthalmologica	not isolated	1.0	0	- systemic antibiotics (not specified) - retrobulbar depomedrol	better VA
2	Davidson, Trans Am Ac Ophthalmol	*Nocardia Asteroides*	N/A	–	- systemic steroids - topical steroids - enucleation	enucleated
3	Fleming, Can J Ophthalmol	*Candida Albicans*	N/A	–	systemic antibiotics (not specified)	N/A
4	Naidoff, Am J Ophthalmol	*Aspergillus Fumigatus*	2.7	–	- intravenous amphotericin - intravitreal amphotericin - topical steroid - enucleation	enucleated
5	Hiss, Ophthalmology	*Criptococcus Neoformans*	0.5	2.7	- intravitreal amphotericin - intravenous amphotericin - oral 5-fluorocytosine - scleral buckling	worse VA
6	Mamalis, Ann Ophthalmol	*Nocardia Asteroides*	1.4	2.3	intravenous salfadiazine and sulfisoxazole	worse VA
7	Gregor, Retina	*Nocardia Asteroides*	1.9	0.3	- intravenous amphotericin - oral 5 fluorocytosine - intravenous thrimetoprim-sulfametoxazole	better VA
8	Coll, Retina	*S. aureus*	1.9	1.3	intravenous antibiotics for endocarditis (oxacillin and gentamicin)	better VA
9	Webber, British J Ophthalmol	*Pseudomonas A.*	2.3	2.3	- systemic amphotericin and ganciclovir - 1^st^ vitrectomy - SRA drainage + intravitreal amikacin, vancomycin and amphotericin B - intravitreal colomycin - intravenous imipenem - 2^nd^ vitrectomy + lensectomy + silicone oil + encircling	same VA
10	Biswas, Retina	*Mycobacterium TB*	0.5	–	- oral prednisolone - topical steroids - periocular hydrocortisone - 1^st^ vitrectomy + lensectomy - intravitreal cefazolin, gentamicin and dexamethasone - 2^nd^ vitrectomy - ATT - evisceration - ATT - topical steroid + atropine	eviscerated
		*Mycobacterium TB*	2.3	0.6		better VA
11	Jolly, Arch Ophthalmol	*Nocardia Asteroides*	N/A	N/A	intravenous trimethoprim-sulfamethoxazole and amikacin	N/A
12	Yarng, Ophthal Surg Las Im	*Klebsiella P.*	2.3	1.6	- intravenous cefonicid, gentamicin, amikacin and cefazolin - intravitreal cefazolin and amikacin × 6 - 6 x intravitreal dexamethasone - vitrectomy + silicone oil + lensectomy + SRA drainage + buckle	better VA
13	Rimpel, British J Ophthalmol	*Streptococcus Viridans*	2.7	–	- intravenous vancomycin and ceftazidime - 3 x intravitreal vancomycin - evisceratio	eviscerated
14	Lakosha, Retina	*Nocardia Farcinica*	0	2.3	trimethoprim-sulfametoxazole	worse VA
15	Harris, Am J Ophthalmol	*Klebsiella P.*	0.2	0.2	- ampicillin, gentamicin, and metronidazole, then piperacillin-tazobactam, gentamicin, then ceftriaxone, gentamicin, and metronidazole - intravitreal amikacin and vancomycin - vitrectomy + SRA removal + amikacin + lensectomy - topical prednisolone and ciprofloxacin - oral prednisone - 2^nd^ vitrectomy + endolaser	same VA
16	Costen, Eye	*Streptococcus Pyogenes*	0.3; 0.5	0.2; 0.2	- intravenous ceftriazone, amoxicillin, benzylpenicillin, cephradine - oral chloramphenicol, clindamycin, ciprofloxacin	BE better VA
17	Yao, Eur J Pediatr	*Klebsiella P.*	N/A	1.9	- AC irrigation + vitrectomy + intravitreal vancomicyn and amikacin + intravitreal dexamethasone - intravenous ceftriaxone - 2^nd^ vitrectomy + SRA drainage - 3^rd^ vitrectomy + buckle + silicone oil	N/A
18	Yoon, Retina	*Klebsiella P.*	2.3	1.9	- intravitreal amikacin and ceftazidime - 1^st^ vitrectomy + SRA drainage - 2^nd^ vitrectomy +silicone oil	better VA
		*Klebsiella P.*	0.4; 0.7	1.5; 1.4	- BE intravitreal vancomycin and amikacin - vitrectomy + SRA drainage	BE worse VA
19	Bozbeyoglu, Retina	*Nocardia Asteroides*	0.3	2.3	- intravenous amphotericin, cefotaxime, amikacin, trimethoprim–sulfamethoxazole - intravitreal amphotericin	worse VA
20	Shah, Indian J Ophthalmol	*Candida A.*	1.9; 1.9	0.8; 0.8	- BE intravitreal amphotericin - oral itraconazole - topical natamycin	BE better VA
21	Wjjesekera, Eye	*Pseudomonas A.*	0	–	- dexamethasone drops - isoniazid and pyridoxine - intravitreal amikacin and vancomycin - vitrectomy + intravitreal amikacin and vancomycin - oral steroids and oral ciprofloxacin - evisceratio	eviscerated
22	Motley, Retina	*Pseudomonas A.*	0.5	–	- topical prednisolone and ketorolac - vitrectomy + intravitreal vancomycin, ceftazidime, and amphotericin - intravenous ceftazidime and tobramycin - 2 x intravitreal and subconjunctival ceftazidime, tobramycin and vancomycin - 2^nd^ vitrectomy + trans-scleral drainage + silicon oil - 3^rd^ vitrectomy + SRA endoresection + silicone oil - enucleation	enucleated
23	Yu, Am J Neurorad	*Nocardia Asteroides*	2.3	–	- antiviral therapy (not specified) - vitrectomy - enucleation	enucleated
24	Rafiei, Europ J Ophthalmol	*Nocardia Asteroides*	1.9	2.3	- systemic cotrimoxazole, linezolid, ciprofloxacin - topical cycloplegic and steroids	worse VA
25	Dodds, Ocul Imm Inflamm	*Nocardia Farcinica*	1.9	–	- intravenous ceftriaxone, clyndamicin, and fluconazole. Then intravenous trimethoprim -sulfamethoxazole - intravitreal amikacin - vitrectomy + SRA aspiration - oral ciprofloxacin	N/A
26	Yang, Ophthalmol	*Klebsiella P.*	N/A	1.0	- intravenous cephalosporin and aminoglycoside - intravitreal antibiotics	N/A
27	Contreras, Ret Cas Brief Rep	*Candida A.*	0.4; 1.9	0.1; 0.7	intravenous caspofungin	BE better VA
28	Christoforidis, Ret Cas Brief Rep	*Klebsiella P.*	2.3	0.4	- intravitreal vancomycin, ceftazidime and dexamethasone - oral prednisone - topical atropine, prednisolone - vitrectomy + intravitreal vancomycin and ceftazidime - intravitreal ceftriaxone - 2^nd^ vitrectomy + SRA drainage + intravitreal ceftazidime - 3^rd^ vitrectomy + scleral buckle	better VA
29	Li, Int Ophthalmol	*Pseudomonas A.*	2.3	–	- intravenous ticarcillin and clavulanate, gentamicin - topical levofloxacin - enucleation	enucleated
30	Jones, Eye	*Nocardia Asteroides*	2.3	1.6	- intravitreal amikacin and vancomycin - systemic cotrimoxazole, linezolid and ciprofloxacin	better VA
31	Trigui, Int Ophthalmol	*S. aureus*	0.7	0	- intravenous ceftriaxone	better VA
32	Eschle-Meniconi, Surv Ophthalmol	*Nocardia Asteroides*	2.3	0.1	- intravenous ceftriaxone, clarithromycin and trimethoprim-sulfamethaxozole - vitrectomy + retinectomy + SRA aspiration + silicone oil	better VA
33	Gupta, Ret Cas Brief Rep	*Pseudomonas A.*	2.3	1.9	- topical ciprofloxacin and gentamicin, then topical ceftazidime and tobramycin - oral cephalexin - intravenous piperacillin–tazobactam, vancomycin and tobramycin	better VA
34	Peeler, J Neuro-ophthalmol	*S. aureus*	2.3; 0	1; 0	- intravenous rifampin, nafcillin, and gentamicin - intravitreal vancomycin × 2 and ceftazidime	RE: better; LE: same
		*Bacillus*	1.9	2.3	- intravenous vancomycin, cefepime, metronidazole, voriconazole, levofloxacin - intravitreal vancomycin × 2 and ceftazidime × 2 - topical moxifloxacin, prednisolone, atropin	worse VA
35	Eisenberg, Ret Cas Brief Rep	*Nocardia Asteroides*	0.7	0.3	- intravitreal vancomycin and × 2 amikacin- - systemic vancomycin, sulfamethoxazole/trimethoprim and meropenem	better VA
36	Arai, Clin Ophthalmol	*Candida A.*	0; 1.4	1.7; −	- intravenous acetylspiramycin + valganciclovir - LE vitrectomy - imipenem/cilastatin, amikacin and levofloxacin - LE intravitreal ceftazidime, vancomycin and voriconazole - LE exenteratio - systemic fosofluconazole - RE 3 x intravitreal ceftazidime, vancomycin and voriconazole - RE vitrectomy + phaco + silicone oil	RE: worse VA; LE eviscerated
37	Siu, BMJ Cas Rep	*Klebsiella P.*	0	1	- intravenous piperacillin/tazobactam, amoxicillin/clavulanate, ceftriazone - oral ciprofloxacin - intravitreal amikacin and ceftazidime - 1^st^vitrectomy + SRA + silicone oil + intravitreal vancomycin and ceftazidime - encircling band + 2^nd^ vitrectomy + silicone oil + phaco	worse VA
38	Shetty, Indian J Ophthalmol	*Mycobacterium TB*	0.2	0.9	- IV methyl prednisolone, then oral prednisolone - ATT + oral steroids	worse VA
39	Silva, Retina	*Nocardia Cyriacigeorgica*	0	0.1	- vitrectomy + retinotomy + SRA biopsy - systemic TMP-SMX and intravenous ertapenem - × 4 intravitreal amikacin	worse VA
		*Nocardia Farcinica*	1.4	2.3	- 1^st^ vitrectomy + lensectomy + SRA biopsy + intravitreal Vancomycin, amikacin, amphotericin - systemic thrimetoprim.-sulfamethoxazole + oral ciprofloxacin and amikacin - 2^nd^ vitrectomy + silicone oil	worse VA
		*Nocardia Farcinica*	0.7	–	- pyrimethamine, sulfonamide, and folinic acid - vitrectomy + SRA biopsy - intravenous TMP-SMX, ceftriaxone and amikacin - × 3 intravitreal amikacin - enucleation	enucleated
40	Won Jin, Optom Vis Sci	*Klebsiella P.*	0.7; 1.2	0; 3	- intravenous acyclovir, ceftazidime + oral prednisolone - LE vitrectomy + intravitreal vancomycin and ceftazidime - intravenous Ceftazidime - RE intravitreal vancomycin and ceftazidime - oral levofloxacin - intravitreal ceftazidime × 3 RE and ×1 LE	RE: better VA; LE: worse VA
41	Richards, Clin Exp Ophthalmol	*Nocardia Beijingensis*	0.2; 2.3	0.3; 2.3	- oral prednisolone - LE vitrectomy + silicone oil + subretinal biopsy - systemic meropenem, ceftriaxone, trimethoprim-sulphamethoxazole and amikacin - RE 3 x intravitreal amikacin	RE worse VA; LE same VE
42	Tsai, BMC Ophthalmol	not isolated	N/A	0	- vitrectomy + intravitreal ceftazidime and amikacin - intravenous ceftriaxone	N/A
43	Kamath, BMK	*Mycobacterium TB*	1.8	N/A	- ATT (rifampicin, pyrazinamide, isoniazid and ethambutol) - oral steroids	N/A
44	Schlaenm Ret Cas Brief Rep	*Fusarium Solani*	1.9	N/A	- systemic piperaciline, tazobactam, imipenem, voriconazole - vitrectomy + SRA drainage + intravitreal amphotericin and voriconazole - intravenous amphotericin	N/A
45	Venkatesh, Int J Ret Vitr	not isolated	1.9	0.6	- intravenous vancomycin and ceftriaxone - intravitreal vancomycin and ceftazidime - vitrectomy + SRA intralesional vancomicin	better VA
46	Soria, Cas Rep Ophthalmol	*Mycobacterium TB*	1.9	1.9	ATT (isoniazid, rifampin, pyrazinamide, and ethambutol)	same VA
47	Martel, J Fran Ophtalmol	*Klebsiella P.*	0.2	0	- IV ceftriaxone and amikacin, then levofloxacin - intravitreal 13 x ceftazidime and 7 x vancomicin - dexamethasone drops	better VA
48	Ganesh, Indian J Ophthalmol	*Mycobacterium TB*	1.0; 0.3	1.5; 0.2	- ATT (isoniazid, rifampicin, pyrazinamide) + oral steroids - azathioprine + intravenous steroids - vitrectomy	RE worse VA; LE better VA
49	Kimura, Cas Rep Ophthal	*Klebsiella P.*	N/A	3.0; 1.7	- BE intravitreal ceftazidime - intravenous linezolid - LE vitrectomy + silicone oil	N/A
50	Boonsopon, J Med Cas Rep	*Mycobacterium TB*	3.0	–	- isoniazid, rifampicin, pyrazinamide and ethambutol - intravenous amikacin, levofloxacin, oral clarithromycin and para-aminosalicylic acid - intravenous ceftriaxone, oral ciprofloxacin - exenteratio	exenterated
51	Pittenger, BMJ	*S. aureus*	N/A	0	- intravenous vancomycin - intravitreal vancomycin and ceftazidime	N/A
52	Fortun, Ophthal Surg Las	*S. aureus* (all)	N/A	0	- systemic vancomicyn and gentamicin	N/A
			0; 1	0; 0	- intravitreal vancomicyn and foscarnet - systemic trimetoprim-sulphametoxazole	RE same VA; LE better VA
			1	0.4	- intravitreal vancomycin and ceftazidime - systemic vancomycin	better VA
			1.7	1.7	- intravitreal vancomycin, ceftazidime and foscarnet - systemic trimethoprim-sulfamethoxazole and foscarnet	same VA
			0; 1.7	0; 0	- intravitreal vancomycin - systemic vancomycin	RE same VA; LE better VA
			0.5; 1.9	0.6; 1.2	- intravitreal vancomycin and ceftazidime - systemic trimethoprim-sulfamethoxazole and vancomycin	
			N/A; N/A	1.2; 3	- intravitreal vancomycin and ceftazidime - systemic vancomycin - RE vitrectomy + buckling + silicone oil	N/A
53	Oduard, Clin Exp Ophthalmol	*Klebsiella P.* *Klebsiella P.*	1.9	0.5	- intravitreal ceftazidime × 2 and vancomycin - intravenous ceftriaxone - topical prednisolone and phenylephrine - vitrectomy + SRA drainage + silicone oil	better VA
			0.3; 2.3	0.3; 0.6	- BE intravitreal vancomycin and ceftazidime: RE × 4 and LE × 5 - BE intravitreal dexamethasone: RE × 4 and LE × 5 - intravenous ceftriaxone - oral steroid - BE topical steroid - RE 1^st^ vitrectomy - RE 2^nd^ vitrectomy + SRA drainage + silicone oil - LE vitrectomy + SRA drainage + silicone oil	RE same VA; LE better VA
54	Pappuru, Int Ophthalmol	*Mycobacterium TB*	1.8	0.3	- ATT - oral steroids	better VA
55	Bendhe, J Ophthal Inflamm Infect	*Roseomonas Mucosa*	1.9	1.3	- 2 x intravitreal ceftazidime and vancomycin - oral cefotaxime - topical moxifloxacin, tobramycin, homatropine and prednisolone - vitrectomy + SRA drainage + silicone oil	better VA
56	Dutta-Majumder, Ocul Imm Inflamm	*Mycobacterium TB* (all)	0.8	0.5	ATT, topical and oral steroid	better VA
			1.9	1.3	ATT, topical steroid	better VA
			1.5	1.8	ATT, periocular steroid	worse VA
			2.7	0.5	ATT, topical and oral steroid	better VA
			1.9	0.2	ATT, topical steroid	better VA
			2.7	2.7	ATT, topical and oral steroid	same VA
			1.9	1.9	ATT, topical and oral steroid	same VA
			1.5	1.9	ATT, topical and oral steroid	worse VA
			0.8	0	ATT, topical steroid	better VA
			1.9	0.5	ATT, topical and oral steroid	better VA
			0.5	2.7	ATT, topical and periocular steroid	worse VA
			1.9	1.0	ATT, oral steroid	better VA
57	Harvey, BMJ Cas rep	*S. aureus*	2.3	1.9	- intravenous flucloxacillin and ceftriaxone - topical steroid - oral antibiotics (not specified)	better VA
58	Prajapati, BMJ Cas Rep	*S. aureus*	2.3	1.0	- intravenous clindamycin, meropenem, flucloxacillin, ganciclovir - oral pyrimethamine	better VA
59	Zafar, BMC Res Not	*Candida A.*	1.9	N/A	- intravitreal amphotericin - vitrectomy + intravitreal amphotericin - oral voriconazole	N/A
60	Chawla, Middle East Afr J Ophthalmol	*Mycobacterium TB*	1; 1.8	0.3; 1.5	- oral ATT (isoniazid, rifampicin, ethambutol, and pyrazinamide) - topical steroid and cycloplegic	BE better VA
61	Puri, Am J Ophthalmol	*Nocardia Farcinica*	N/A	0.7	- intravenous vancomycin, piperacillin-tazobactam and micafungin - intravitreal amikacin - oral bactrim and augmentin	N/A
62	Xu, BMC Ophthalmol	*Nocardia*	1.9	1.0	- intravitreal vancomycin and ceftazidime - topical levofloxacine and steroids - oral trimethoprim and sulfamethoxazole + oral prednisone	better VA
63	Tran, Clin Exp Ophthal	*Nocardia Farcinica*	0.5	–	- systemic moxifloxacin, voriconazole and amphotericin - intravitreal multiple injections of voriconazole, vancomicin, ceftazidime and foscarnet - evisceratio	eviscerated
64	Scavelli, Am J Cas Rep	*Nocardia Farcinica*	1.9	0.3	- systemic sulfamethoxazole/trimethoprim and imipenem - intravitreal amikacin × 4	better VA
65	Manoharam, JRSM Open	*Proteus Mirabilis*, *Enterococcus Faecium*, *E. coli*	1.9	1.9	- intravitreal vancomicin × 2, ceftazidime × 2 - topical antibiotics, steroid and cycloplegic drops - oral antibiotics (not specified) - intravenous linezolid, meropenem and fluconazole	same VA
66	Mohd-Ilham, Cureus	*Klebsiella P.*	1.0	0.8	- intravenous cefepime and ciprofloxacin - intravitreal vancomycin and ceftazidime - topical cefuroxime, gentamicin and dexamethasone) - vitrectomy + silicone oil	better VA
67	Angermann, Ocul Imm Inflamm	*Nocardia Cyriacigeorgica*	N/A	N/A	- vitrectomy + SRA biopsy - systemic trimethoprim-sulfamethoxazole	N/A
3.1.4.68	Dogra, Ocul Imm Inflamm	*Klebsiella P.*	1.9	0.8	- intravitreal vancomycin and ceftazidime - topical moxifloxacin, prednisolone and cycloplegics - intravenous piperacillin/tazobactam - intravitreal piperacillin/tazobactam + dexamethasone	better VA
69	Yiesiltas, Ocular Imm Inflamm	*Candida A.*	1.9	1.9	- oral methylprednisone + co-trimoxazole - intravenous amphotericin - topical steroid and cyclopentolate - intravitreal voriconazole and amphotericin - oral fluconazole - vitrectomy + silicone oil	same VA
70	Shen, Retina	*Klebsiella P.*	2.3	2.3	- intravitreal vancomycin	same VA
71	Hojjatie, J Ophthalmic Inflamm Infect	*Nocardia Farcinica*	1.2	0.7	- intravitreal voriconazole, vancomycin and amikacin - topical steroids and cycloplegics - vitrectomy	better VA
72	Vamsidhar, J R Coll Physicians Edinb	*S. aureus*	1.3; 1.8	0.5; 0.5	- intravenous ceftazidime, vancomycin and cloxacillin - oral ATT - oral cloxacillin	BE better VA
73	Lim, Case Rep Ophthalmol Med	*Klebsiella P.*	2.3	1.0	- intravenous ceftriaxone, metronidazole and amikacin - intravitreal vancomycin × 1, ceftazidime × 9 and dexamethasone × 4 - topical antibiotics - vitrectomy + intravitreal ceftazidime	better VA
		not isolated	1.9	2.3	- oral moxifloxacin, intravenous ceftriaxone, amikacin and metronidazole - intravitreal vancomicin, ceftazidime, ceftriaxone and dexamethasone - vitrectomy + intravitreal ceftriaxone and vancomycin	better VA
74	Nair, Indian J Ophthalmol	*Mycobacterium TB*	1.9	0.4	- oral ATT (isoniazid, rifampicin, ethambutol and pyrazinamide) - steroids	better VA
75	Kapoor, Indian J Ophthalmol	not isolated	2.7	1.0	- intravenous ceftriaxone - intravitreal vancomicin, piperacillin and amphotericin - vitrectomy	better VA
76	Malik, BMJ Cas Rep	*Nocardia*	0.7	0.7	- intravitreal amikacin, vancomicin and amphotericin - intravenous amikacin and imipenem - oral trimethoprim-sulfamethoxazole and linezolid - vitrectomy	same VA
77	Fan, Ret Cas Brief Rep	*Klebsiella P.*	2.3	N/A	- vitrectomy + phaco + AC washout + intravitreal ceftazidime, vancomicine and amikacin - intravenous vancomicin and cefepime - enucleation	enucleated
78	Cunha, Ret Cas Brief Rep	*Nocardia Abscessus*	0.4	0.3	- systemic trimetoprim-sulfalethoxazol, imipenem and cefipime - intravitreal bevacizumab	better VA

*S/N* Study number, *RE* Right eye, *LE* Left eye, *BE* Both eyes, *M* Male, *F* Female, *SRA* Subretinal abscess, *N/A* Not available

## Discussion

Our review of the literature showed that *Nocardia* was the most frequent causative pathogen of SRA associated with systemic infective foci, while for SRA in absence of systemic foci *Aspergillus* was seen with a higher frequency. In absolute numbers *Nocardia* was the most frequent causative agent.

In SRA without systemic infective foci the pathogen was more commonly isolated from SRA if there was no vitreous involvement, while for forms with vitreous involvement a similar yielding rate from vitreous and from SRA was observed. By contrast, in presence of a systemic infective focus the pathogen was isolated mainly from extra-ocular sites, and when ocular sampling was performed in cases of SRA with no vitreous involvement the observed yielding rate for vitreous was 20% and for SRA was 100%. In the forms with vitritis where ocular sampling was done the vitreous was the commonest ocular site of pathogen identification. However, failure of vitreous sampling in isolating the pathogen has been described by many authors, who were subsequently able to isolate it by direct drainage of the lesion [11, 19]. Despite vitritis being observed with a similar frequency in both groups (81.8% versus 83%) the vitreous yielding rate was higher in the forms without systemic infective foci (43.8% versus 35.7%).

The most common systemic predisposing conditions of SRA were immunosuppression and diabetes mellitus, the former being more frequent in SRA without systemic foci (35% of patients) and the latter being more frequent in SRA associated with infective foci (25.5% of patients). Isolated SRA was more frequently observed in cases without systemic infection (18.2% of eyes versus 10.8% of eyes of group 2). A higher frequency of bilateral involvement was observed in the forms with systemic foci, where it was detected in 17.6% of patients (30% of eyes) compared to 10% of patients (18.2% of eyes) without systemic foci.

Reduced vision was observed with a similar frequency in both groups (70% and 72.5%). Baseline visual acuity did not show a significant difference between the groups, but final visual acuity was better in the group associated with systemic foci (*p* = 0.003).

*Pseudomonas*, *Nocardia* and *Aspergillus* were the microorganisms related to a worse prognosis requiring enucleation or evisceration. Some of the cases with poor prognosis may be related to delay in diagnosis and management or to systemic factors as immunosuppressive medication intake that may have an impact on the natural history of the disease.

No standard approach exists for the management of SRA because, unlike endophthalmitis, no guidelines are available at present and there is no consensus on the various proposed therapeutic approaches. Systemic and intravitreal antibiotics/antifungals and vitrectomy are the mainstay treatment in the majority of cases, but there is no consensus on the timing of vitrectomy, which in fact differed between the two groups: in group 1 vitrectomy was performed with similar frequency as first or second line treatment, while for group 2 it was mostly performed when previous non-surgical treatments failed. While for endogenous endophthalmitis the standard of care includes vitreous biopsy and intravitreal antibiotics combined with systemic antibiotics and oral steroids (once fungal infection has been ruled out), for SRA there is no universal approach. In case of no vitreous involvement a prompt systemic treatment can achieve an excellent prognosis, but a close follow-up is essential to identify the potential progression of SRA into the vitreous cavity, needing immediate revision of the therapeutic strategy. The effectiveness of intravitreal antibiotics, including vancomycin for Gram positive and ceftazidime for Gram negative bacteria, is controversial as they may not fully penetrate into the subretinal space. Surgical treatment of SRA, including pars plana vitrectomy and abscess drainage, is considered a second-line therapy when conservative treatments are not effective. However, for very aggressive pathogens some authors advocate an early surgical intervention. Some authors adopted the technique of intralesional antibiotics, namely injection of antibiotics into the subretinal space through a small retinotomy. Compared to abscess drainage, the technique has the advantage of carrying a lower risk of retinal detachment [20, 21], but in isolated SRA it could favor the invasion of the vitreous cavity by the pathogen. Tsai and Peng suggested that if the SRA is smaller than four disc areas, pars plana vitrectomy with intravitreal antibiotic injection could be successful, whereas, in larger lesions, vitrectomy with retinectomy to remove the abscess should be considered [22]. Internal drainage of the SRA leads to resolution of the infection, but carries the risk of postoperative retinal detachment due to proliferative vitreoretinopathy and therefore should be considered in cases that fail to respond to conventional therapy. Eschle-Meniconi et al. suggested fluorescein angiography as a guide for the management strategy as it helps understanding which layer is affected by the infection and identify a potential early invasion of the vitreous: if at presentation a late leakage of the lesion is observed, it means that the retinal pigmented epithelium is disrupted and the vitreous is affected and then vitrectomy and subretinal biopsy should be performed in the first instance. If no late leakage is seen, the infection is at an initial stage and limited to the subretinal space and a trans-vitreal fine needle biopsy or vitreous tap, intravitreal antibiotics and systemic treatment are the preferred options to start with. If the patient is already under treatment for an infection site elsewhere, then a late leakage on angiography would indicate the need for intravitreal antibiotics in case of small and peripheral SRA or for vitrectomy for larger ones [23]. However, vitreous involvement in practice can be identified by clinical assessment and multimodal imaging with no need for invasive tests.

## Conclusion

Although SRA can develop even in the absence of clinically detectable systemic infectious foci it is of primary importance to perform a prompt physical examination and systemic investigations in order to identify or rule out a source of infection elsewhere or masquerading conditions. Our review showed that no universal approach exists for SRA. Systemic broad-spectrum antibiotics are of primary importance and should be used in all cases of SRA, even in the absence of vitreous involvement and of identifiable infective foci, given the high risk of an undiagnosed underlying systemic infection.

## Data Availability

Not applicable.
